# Enhancing self-management in chronic pain: Reflections on a qualitative study

**DOI:** 10.1080/24740527.2024.2448997

**Published:** 2025-01-24

**Authors:** John Patrick C. Toledo

**Affiliations:** Department of Theology and Religious Education, De La Salle University, 2401 Taft Ave, Malate, Manila, Metro Manila 1004, Philippines

Dear Editor,

I’ve read with interest the qualitative study titled “Discharge Doesn’t Mean the End: Exploring Success in Discharge to Community Self-Management for Young Adults Living with Chronic Pain,” authored by Souraiya Kassam et al.^1^ The study explores the complex nature of successful discharges and offers important insights into the experiences of young adults moving from specialized chronic pain treatments to community self-management.

The study’s strong qualitative approach, which used semistructured interviews to let participants share their experiences in their own words, is one of its strongest features. This approach captured the complexity of participants’ emotional and practical challenges during their transition, which quantitative studies often overlook.^1^ The findings reveal that effective discharge involves a collaborative process between health care providers and patients, highlighting the necessity for personalized self-management plans tailored to individual needs.^1^

Further, the study has an abundance of potential; that is, increasing the number of participants may improve the findings’ applicability to various groups of young people with chronic pain. Additionally, incorporating longitudinal studies could provide a clearer picture of the long-term outcomes of discharge planning on users’ health and well-being.^1^

By highlighting the practical consequences of efficient discharge planning, the work makes a substantial scientific contribution to public discourse. Its observations highlight how crucial it is to incorporate community resources into medical procedures to improve self-management.

On a personal note, this letter to the editor suggests creating patient-centered self-management plans that include ongoing tools and support, which are crucial for empowering young adults throughout their health care journeys. For young adults with chronic pain, these programs are essential for promoting favorable health outcomes and increasing their general quality of life.
